# Echocardiographic Assessment of Left Atrial Mechanics in Patients with Atrial Fibrillation Undergoing Electrical Cardioversion: A Systematic Review

**DOI:** 10.3390/jcm13216296

**Published:** 2024-10-22

**Authors:** Andrea Sonaglioni, Gian Luigi Nicolosi, Antonino Bruno, Michele Lombardo, Paola Muti

**Affiliations:** 1 Division of Cardiology, IRCCS MultiMedica, 20123 Milan, Italy; michele.lombardo@multimedica.it; 2 Division of Cardiology, Policlinico San Giorgio, 33170 Pordenone, Italy; gianluigi.nicolosi@gmail.com; 3 Laboratory of Innate Immunity, IRCCS MultiMedica, 20138 Milan, Italy; antonino.bruno@multimedica.it; 4 Laboratory of Immunology and General Pathology, Department of Biotechnology and Life Sciences, University of Insubria, 21100 Varese, Italy; 5 Department of Biomedical, Surgical and Dental Sciences, Università di Milano, 20122 Milan, Italy; pmuti26@gmail.com; 6 Scientific Direction, IRCCS MultiMedica, 20138 Milan, Italy

**Keywords:** atrial fibrillation, electrical cardioversion, left atrial mechanics, left atrial strain, prognostic risk stratification

## Abstract

**Background**: To date, only a few studies have evaluated left atrial (LA) mechanics in patients with atrial fibrillation (AF) scheduled for electrical cardioversion (ECV). The present systematic review has been primarily designed to summarize the main findings of these studies and to examine the overall effect of AF on left atrial reservoir strain (LASr) in patients undergoing ECV. **Methods**: All the echocardiographic studies evaluating the effect of AF on LA mechanics in patients scheduled for ECV, selected from the PubMed and EMBASE databases, were included. There was no limitation of time period. The risk of bias was assessed by using the National Institutes of Health (NIH) Quality Assessment Tool for Observational Cohort and Cross-Sectional Studies. **Results**: The full texts of 12 studies with 880 AF patients were analyzed. The pooled ECV success rate was 91.5% (range 65.8–100%). Over a median follow-up of 5.4 months (range 0.3–12 months), 35.2% of the patients (range 5–68.8%) experienced AF recurrence. At baseline, the average LASr was 11.4% (range 6.2–17.7%). A reduced LASr before ECV was strongly correlated with reduced left atrial appendage (LAA) flow velocities and/or thrombosis. The main independent predictors of cardioversion failure were impaired LASr and previous AF history. A severe LASr deterioration was independently correlated with AF recurrence after ECV. The other independent predictors of AR relapses were LA asynchrony, reduced difference between post- and pre-ECV LASr, and reduced right atrial reservoir strain. **Conclusions**: LASr assessment before ECV may provide useful prognostic information about AF relapses and improve the refinement of the thromboembolic risk of AF patients scheduled for ECV.

## 1. Introduction

Atrial fibrillation (AF) is the most common clinically significant cardiac rhythm disorder worldwide [1] and is a major risk factor for stroke and heart failure [2]. Most AF patients develop left atrial (LA) enlargement. The increased LA size, a marker of atrial structural remodeling, has been found to be an independent predictor of AF recurrences after successful electrical cardioversion (ECV) or radiofrequency ablation [3,4,5]. Although the strong relationship between LA dilatation and AF is widely recognized, it is not clear whether LA enlargement is the consequence or the cause of the arrhythmia [6,7]. Moreover, some authors have hypothesized that LA enlargement occurs only if AF becomes chronic [8,9]. It is noteworthy that subtle functional alterations may be present in AF patients with normal LA size on transthoracic echocardiography (TTE). Recent evidence indicates that LA enlargement is anticipated by the impairment in LA strain [10,11,12]. This innovative biomarker of LA function may be easily assessed by speckle tracking echocardiography (STE), a semi-automated and angle-independent technique [13]. The implementation of LA morphological assessment with LA strain analysis by strain echocardiographic imaging may provide more comprehensive information on LA mechanics. The incremental prognostic role of LA strain during the reservoir phase (LASr) over LA size as a predictor of new-onset AF [14,15] or AF recurrences [16,17] has been consistently reported in various study groups. To date, the majority of authors have investigated LA strain in AF patients who previously underwent a successful ECV during the follow-up period, whereas only a few studies have evaluated LA mechanics in AF patients undergoing ECV before the planned procedure. The present systematic review has been primarily designed to summarize the main findings of these studies and to examine the overall effect of paroxysmal and/or persistent AF on LA mechanics in patients planned for ECV. The pathophysiological mechanisms underpinning LASr impairment in these patients will be discussed as well.

## 2. Materials and Methods

This systematic review was performed according to the Preferred Reporting Items for Systematic Reviews and Meta-analyses (PRISMA) guidelines [18] and was registered in the INPLASY database on 8 September 2024 (registration number INPLASY202490037).

### 2.1. Search Strategy

A comprehensive search of all the echocardiographic studies evaluating the effect of paroxysmal and/or persistent AF on LA mechanics in patients planned for ECV was carried out by two independent reviewers (A.S. and M.L.) between August and September 2024 by using the Medline and EMBASE databases. The search was last updated on 30 September 2024. The search strategy included the following terms: “atrial fibrillation” AND “electrical cardioversion” AND “left atrial mechanics” OR “left atrial reservoir strain” OR “left atrial appendage strain” AND “speckle tracking echocardiography” AND “two-dimensional transthoracic echocardiography” OR “three-dimensional transthoracic echocardiography” AND “transesophageal echocardiography”. The search was limited to full-text articles published in English, whereas non-English language studies were excluded. There was no time frame restriction for the inclusion of studies.

### 2.2. Eligibility Criteria

All the echocardiographic studies evaluating the effect of paroxysmal and/or persistent AF on LA mechanics in patients undergoing ECV, regardless of the time frame, were included. Conversely, imaging studies conducted on AF patients who previously underwent ECV, echocardiographic studies conducted on AF patients scheduled for ECV without LA mechanics assessment, studies involving patients aged <18 years non-clinical articles, animal studies, duplicate articles, case reports, conference presentations, reviews, correspondences, editorials, letters without data, and abstracts were excluded.

### 2.3. Study Selection and Data Extraction

Two reviewers (A.S. and M.L.) screened the databases according to the inclusion criteria and performed data extraction independently. The following information concerning AF patients: (1) demographics (age and sex); (2) anthropometrics (body mass index); (3) prevalence of the most common cardiovascular risk factors (hypertension, smoking, type 2 diabetes mellitus, and dyslipidemia); (4) previous history of coronary artery disease (CAD), transient ischemic attack (TIA)/stroke, and/or heart failure; (5) AF duration, heart rate at echocardiographic assessment, CHA2DS2-Vasc (Congestive heart failure or left ventricular dysfunction, Hypertension, Age ≥ 75 years, Diabetes, Stroke/TIA, Vascular disease, Age 65–74 years, and Sex category) Score; (6) laboratory data, such as serum levels of brain natriuretic peptide (BNP) or N-terminal pro-brain natriuretic peptide (NT-proBNP) and C-reactive protein (CRP); (7) 2D-TTE parameters, including cardiac chamber cavity size, left ventricular filling pressures (LVFPs) expressed by the E/e’ ratio, biventricular systolic function assessed by left ventricular ejection fraction (LVEF), and tricuspid annular plane systolic excursion (TAPSE), respectively, and pulmonary hemodynamics; (8) 2D-transesophageal echocardiography (TEE) parameters, such as left atrial appendage (LAA) flow velocities and the eventual presence of LAA thrombosis (LAAT); (9) STE indices, particularly peak systolic LA and left atrial appendage (LAA) strain and strain rate (SR) values; (10) current medical treatment; and (11) ECV results and follow-up data were independently collected by the two reviewers. Possible discrepancies between the reviewers were resolved through a consensus discussion with the involvement of a third author (G.L.N.), who checked the extracted data to ensure accuracy, completeness, and consistency.

### 2.4. Left Atrial Reservoir Strain Assessment

LASr is obtained by manually tracing the LA endocardial border in the apical 4-chamber view (“monoplane” method) or in both apical 4- and 2-chamber views (“biplane” method), thus delineating a region of interest (ROI), composed of 6 or 7 segments, depending on the software configuration. Then, after the eventual manual adjustment of the ROI, the longitudinal strain curves are automatically generated by the software for each atrial segment. LASr, measured at the end of the reservoir phase, is calculated by averaging the values observed in all the LA segments [19]. Given that in AF, the pump function is lost, only LASr can be measured, whereas peak LA diastolic strain (the expression of the LA contractile function) is not determined. LASr is generally calculated as the mean of 3–5 consecutive measurements. The additive measures of LA mechanics are the standard deviation of time-to-peak strain (TP-SD), computed as the SD of the time to the maximal deformation of each strain curve, and the dispersion of time-to-peak longitudinal strain (dTP-LS), calculated as the maximal difference in the TPLS of the 12 atrial segments (6 LA segments from the 4-chamber view and 6 segments from the 2-chamber view) [20]. Large values of TP-SD or dTP-LS are suggestive of a high grade of LA asynchrony in AF patients.

### 2.5. Risk of Bias Assessment

The articles included in this systematic review were assessed for risk of bias (RoB) using the National Institutes of Health (NIH) Quality Assessment Tool for Observational Cohort and Cross-Sectional Studies [21]. All the studies were assigned a “yes”, “no”, or “other” to each of the 14 criteria outlined in the appraisal tool. Notably, for each study included in the present systematic review, when the single criterion was satisfied, we assigned a “yes”; when it was not satisfied, we assigned a “no”; and when it was not evaluated, we assigned a “not specified (NS)”. Then, by considering each criterion, the investigators evaluated the overall quality of the study and assigned an overall “good” (met 11–14 criteria), “fair” (met 6–10 criteria), or “poor” (met 0–5 criteria) rating to each study. The quality rating was independently estimated by two authors (A.S. and G.L.N.). Disagreement was resolved by consensus.

**Figure 1:**
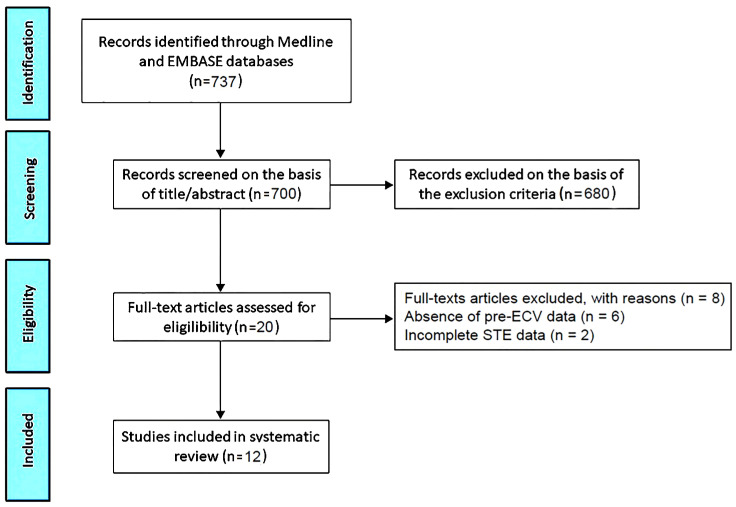
Flow diagram used for identifying the included studies. ECV, electrical cardioversion; STE, speckle tracking echocardiography.

The PRISMA flow diagram used for identifying the included studies is depicted in Figure 1.

## 3. Results

The initial search yielded a total of 737 studies. Of those, 37 (5%) were removed as duplicates. After screening titles and abstracts, a further 680 studies (92.3%) were removed on the basis of the exclusion criteria. The evaluation of the full text of the remaining 20 studies (2.7%) resulted in a further 8 exclusions (1.1%). A total of 12 studies (1.6%) [22,23,24,25,26,27,28,29,30,31,32,33] were thus included in this systematic review, totaling 880 AF patients.

The clinical characteristics and main findings of the included studies are summarized in Table 1.

The included studies were published between 2008 and 2023. Five studies were performed in Italy, and one in Turkey, the USA, Poland, Spain, France, Sweden, and Germany. Nine studies (75% of the total) had a prospective design, whereas the remaining three (25% of the total) were retrospective.

Concerning LA strain assessment, the great majority of studies (83.3% of the total) performed two-dimensional (2D)-TTE implemented with STE analysis, whereas only two studies (16.7% of the total) measured LA strain parameters by three-dimensional (3D)-STE. Ten studies analyzed myocardial strain parameters by using the General Electric (GE) software (Vivid 7 or 9), while the remaining two studies used the Philips software (EPIQ or Sparq system).

The pooled success rate of ECV was 91.5%, ranging from 65.8% to 100%. During a median follow-up of 5.4 months (range 0.3–12 months), 35.2% of the patients (range 5–68.8% of total) experienced AF recurrence.

Table 2 reports all the baseline demographic, anthropometric, clinical, laboratory, and echocardiographic parameters collected by the included studies in AF patients scheduled for ECV.

The AF patients examined by the included studies had a mean age of 66.9 years (range 55–72 years) and were predominantly males (64.6% of total). The prevalence of hypertension and dyslipidemia among the AF patients was 65.1% and 48.7%, respectively, whereas smoking and type 2 diabetes were less frequently detected, in 25.9% and 19.5% of the AF patients, respectively. The average CHA_2_DS_2_-Vasc score was 2.6 (range 0.7–3.8), indicating a high risk for thromboembolic complications. The estimated mean AF duration was 2.9 months (range 0.03–5.7 months). The average heart rate on electrocardiogram (ECG) was 97 bpm (range 81–113 bpm).

The three studies that analyzed biochemical parameters in AF patients before ECV showed a moderate increase in the serum NT-proBNP levels and a mild increase in the serum CRP levels.

On conventional TTE, the AF patients were found with mild-to-moderate LA enlargement, normal biventricular cavity size, and mild biventricular systolic dysfunction, as assessed by LVEF (average value < 55%) and TAPSE (average value < 20 mm), respectively; the E/e’ ratio (average value < 13) excluded elevated LVFPs; and finally, the systolic pulmonary artery pressure (average value > 35 mmHg) was slightly increased.

With regard to TEE parameters, a mild reduction in the average LAA emptying and filling velocities was documented in AF patients by three studies, whereas LAAT was reported in 11.6% of the AF patients (range 7.1–16%) by two studies.

LASr was calculated with a “biplane method” by seven studies (58.3% of the total), while in the remaining five studies (41.7% of the total), LASr was obtained from the apical four-chamber view (“monoplane” method). At baseline, regardless of the method employed for LA mechanics assessment (biplane or monoplane), the AF patients were uniformly diagnosed with a severe peak systolic LASr impairment in comparison to the accepted reference values (≥39%) [34,35]. Notably, the average LASr value was 11.4% (range 6.2–17.7%).

Intra- and inter-rater variability analysis for LASr assessment, expressed as intraclass correlation coefficients with their 95% confidence intervals, was performed by eight studies (66.7% of the total). The reproducibility of atrial strain measurements was fair-to-good in one study [31] and good-to-excellent in the remaining seven studies.

Some authors identified, among the AF patients before ECV, an increased LA asynchrony, quantified as large values of TP-SD [23,24] or dTP-LS [26], that significantly improved after a successful ECV.

The two studies that evaluated LAA deformation in AF [22,23,24,25,26,27,28,29,30] observed a significant attenuation of the peak systolic LAA strain and SR values. A concomitant impairment of right atrial reservoir strain (RASr) was detected by two authors [29,33].

A strong linear correlation between LASr and LAA emptying velocities [22], between LA peak-to-peak SR and both LAA filling and emptying velocities [30], and between LASr and LAA systolic strain and SR values [22,23,24,25,26,27,28,29,30] was demonstrated. LASr/left atrial volume index (LAVi) < 0.08 [27], LASr < 8.1% [28] or ≤9.1% [30] showed the highest sensitivity and specificity for predicting reduced LAA emptying velocities and/or LAAT in AF patients undergoing ECV. A prolonged atrial stunning following ECV was reported in the patients with previous AF history [31] and in the patients with heart failure with preserved ejection fraction (HFpEF) [32].

**Figure 2:**
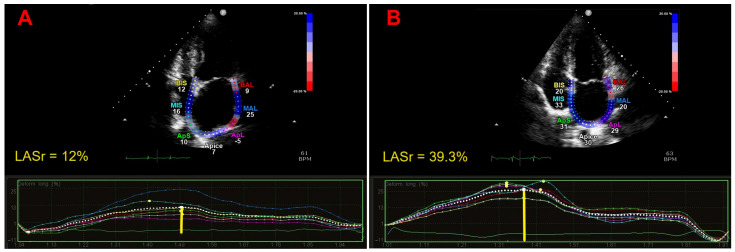
Representative examples of LASr calculation from the apical 4-chamber view in a patient with persistent AF of greater than 3 months’ duration and previous AF history planned for ECV (Panel (**A**)), and in a healthy individual without AF (Panel (**B**)). The longitudinal strain curves of the seven atrial segments are depicted with different colors. The dotted line indicates the average atrial longitudinal strain. The yellow line indicates the LASr magnitude. In the present case, the peak systolic LASr obtained in the patient with AF duration > 6 months and previous AF history was severely impaired in comparison to the healthy control without AF and to the accepted reference values. Moreover, the nonhomogeneous distribution of the regional atrial strain curves indicated significant LA asynchrony in AF patients. AF, atrial fibrillation; ECV, electrical cardioversion; LA, left atrial; LASr, left atrial reservoir strain.

Figure 2 illustrates the representative examples of LASr calculation from the apical four-chamber view in a patient with persistent AF of greater than 3 months’ duration and previous AF history planned for ECV (Panel A), and in a healthy individual without AF (Panel B).

Overall, in the AF patients who were successfully cardioverted, ECV determined a significant decrease in bi-atrial volumes [29] and a concomitant improvement in LASr [32], LAA strain [22], and RASr [29], leading to reverse bi-atrial remodeling.

ECV failure was independently predicted by reduced LASr in absolute value and in relation to LAVi [27], and previous AF history [31], whereas the prior use of statins was found to be a predictor of a successful ECV [26].

The included studies also showed a prognostic risk stratification for AF recurrence in the AF patients who underwent ECV. The main independent predictors of AF relapse after ECV over follow-up period were (1) LA asynchrony, quantified as increased TP-SD of regional atrial strains [23,24] or TP-LS [26] before ECV; (2) reduced difference between post- and pre-ECV LASr [25,33]; (3) previous AF history [31]; (4) reduced LASr (cut-off ≤ 10%) and RASr magnitude (cut-off ≤ 15%) at baseline [33].

Information regarding the current medical treatment was provided by a percentage of studies ranging from 16.7% to 58.3% of the total. At baseline, two-thirds of the AF patients were treated with oral anticoagulants and approximately half of the patients received angiotensin-converting enzyme inhibitors (ACEi) or angiotensin receptor blockers (ARBs), beta blockers, and diuretics, while one-third of the total were prescribed with antiplatelets, calcium channel blockers, digoxin, antiarrhythmic drugs, and statins.

With regard to the RoB assessment, the NIH quality rating was estimated as fair for six studies and good for six studies (Table 3). The Cohen’s Kappa coefficient for the agreement between the reviewers in the RoB assessment was interpreted as a substantial agreement, k = 0.80.

## 4. Discussion

### 4.1. Main Findings of the Present Systematic Review

The present systematic review, which analyzed 12 studies performed over a 15-year period, highlighted the high feasibility and reproducibility of strain echocardiographic imaging for assessing LA mechanics in AF patients planned for ECV. LA function was predominantly assessed by using a biplane method, with low inter-vendor variability (two vendors). AF patients undergoing ECV were found with a mild LA enlargement on 2D-TTE and a significant LASr impairment on STE analysis. The procedure was effective in more than 90% of the cases. However, approximately one-third of the AF patients experienced AF recurrence over a median follow-up of 5.4 months.

The included studies demonstrated the incremental diagnostic and prognostic role of LASr assessed by strain echocardiographic imaging in AF patients scheduled for ECV for noninvasively detecting a significant dysfunction of the atrio-auricular complex and for predicting AF recurrences over the follow-up period. Indeed, LASr magnitude was found to independently correlate with LAA flow velocities and LAA strain, and to independently predict ECV success and AF recurrences after ECV: the lowest was the pre-ECV LASr value, and the highest was the pro-thrombotic risk, the rate of cardioversion failure, and the probability of AF relapse during a short- to mid-term follow-up.

### 4.2. Pathophysiological Mechanisms of Atrial Structural Remodeling

AF patients generally develop an LA structural remodeling, characterized by the progressive increase in the collagen content and a decrease in atrial myocytes within the atrial wall [36]. This process produces LA fibrosis, dysfunction, and enlargement, initiated and maintained by the cardiovascular risk factors and cardiovascular disease burden and strongly correlated with AF duration [37,38]. The increase in collagen fibers constitutes electrical barriers, causing delayed electromechanical conduction and the asynchronous propagation of electrical activation, which contributes to the sustainability and persistence of atrial arrhythmia [39,40,41].

LASr is the most important marker of the structural atrial remodeling. It is inversely correlated with the degree of myocardial fibrosis, as demonstrated in cardiac magnetic resonance (CMR) studies [42,43]. Thus, a reduced LA strain is a marker of fibrous atria with decreased contractile capacity [44].

Due to the strong correlation between LA and LAA function [22,30], LASr impairment may indirectly reflect low LAA flow velocities and reduced LAA deformation on TEE, potentially identifying, among AF patients, those with increased thromboembolic risk [45]. Indeed, the replacement of healthy atrial tissue with fibrotic tissue in AF can lead to reduced atrial contractile function and blood stasis, thus contributing to the process of thrombus formation [45,46,47].

The significant impairment of LASr in AF patients undergoing ECV, detected by the included studies, was in alignment with the published literature [48,49,50].

It is noteworthy that the AF-related structural remodeling may also affect the right atrium [51,52]. The two echocardiographic studies that evaluated RASr in AF patients planned for ECV confirmed that persistent AF was associated with bi-atrial enlargement and bi-atrial dysfunction [29,33].

The increased serum NT-proBNP levels observed in AF patients by 25% of the included studies were likely related to the atrial wall stress secondary to the increased LVFPs [53,54]. Arvanitis P et al. [31] demonstrated that interleukin-6, high-sensitivity cardiac-troponin-T, NT-proBNP, prothrombin fragment 1 + 2, and fibrinogen decreased significantly after effective ECV, indicating that the increase in all these inflammatory, cardiac, and coagulation biomarkers was a direct consequence of AF [55,56,57,58].

### 4.3. Atrial Reverse Remodeling

The restoration of sinus rhythm after a successful ECV was associated with the recovery of LA mechanical synchrony (as expressed by TP-SD decrease) [24] and bi-atrial reverse remodeling, characterized by the gradual decrease in the bi-atrial size and the concomitant progressive increase in the bi-atrial reservoir strain [25,29]. The increased values of LASr after effective ECV resulted from improved LA filling; however, as previously demonstrated [49,59], the mean LASr did not normalize completely after a successful ECV. This process, defined as ‘‘atrial stunning”, is characterized by the impairment of LA mechanics, which reaches a maximum immediately after ECV and improves progressively within 4 to 6 weeks; however, this phenomenon is highly variable depending on the AF duration, the atrial size, and the coexistence of structural heart disease [25,48]. Notably, atrial stunning has been found to be prolonged in HFpEF patients [32]. Moreover, some authors have reported that LA contractility can remain modestly impaired until a 3-year follow-up period [60,61].

Structural atrial reverse remodeling, primarily characterized by a significant reduction in LA size, may also be quantified by other noninvasive imaging modalities, such as computed tomography [62] and CMR [63].

Antihypertensive drugs, particularly ACEi or ARBs and spironolactone, may promote atrial reverse remodeling, resulting in a significant LA size reduction and decreased amount of fibrosis [64,65]. Nonmedical interventions, such as dietary modification, physical activity, and weight loss, may also contribute to a reduction in LA size and improvement in LA function [66].

### 4.4. Predictors of Cardioversion Failure or Success

The high ECV success rate detected by the included studies was consistent with the literature data [67,68]. Cardioversion failure was predicted by reduced LASr in absolute value and in relation to LAVi [27]. This finding would suggest that a more extensive LA fibrotic remodeling negatively affects ECV results. Another factor associated with an unsuccessful ECV was the previous AF history [33]. A plausible explanation for the ECV unsuccess in patients with a prior AF history is a slower reverse remodeling likely related to irreversible structural remodeling occurring after previous episodes of AF [69,70].

Other previously reported predictors of ECV failure are male gender [71], advanced age [72], high body weight [73] and high body surface area [74], diabetes [75], low estimated glomerular filtration rate [76], CHA_2_DS_2_-VASc score > 2 [77], left ventricular systolic dysfunction [78], larger LAVi [3], increased LVFPs [79], and AF duration > 3 months [80].

Consistent with the literature data [81,82,83], AF duration < 3 months, pre-treatment with amiodarone, and the prior use of statins were found by the included studies to be independently associated with ECV success. The favorable effect of statins in limiting AF recurrences could be attributed to their pleiotropic properties [84]. ECV success may also be expected in patients with paroxysmal AF [85], flutter rhythm [86], young age [86], and when using biphasic waveform [85].

### 4.5. Predictors of AF Recurrence

The present systematic review confirmed that LAVi, a conventional TTE-derived index of LA size, did not improve the identification of patients at risk for AF recurrence after ECV [87]. Indeed, it was not associated with the recurrence of AF after elective ECV [33]. The lack of predictive power of LAVi in AF patients scheduled for ECV could be explained by the superior ability of LASr to detect the subclinical impairment of the LA myocardium, regardless of LA size [88]. Differently from LAVi, a low pre-ECV LASr magnitude was found to be independently associated with AF recurrence over follow-up. This finding was also observed by a number of echocardiographic studies that assessed LASr after a successful ECV in AF patients who maintained sinus rhythm vs. those who experienced AF recurrence over a follow-up period, not included in the present systematic review [89,90,91,92,93,94]. Importantly, the reduced difference between post- and pre-ECV LASr was measured in AF patients who experienced AF relapses after ECV [25,33].

**Figure 3:**
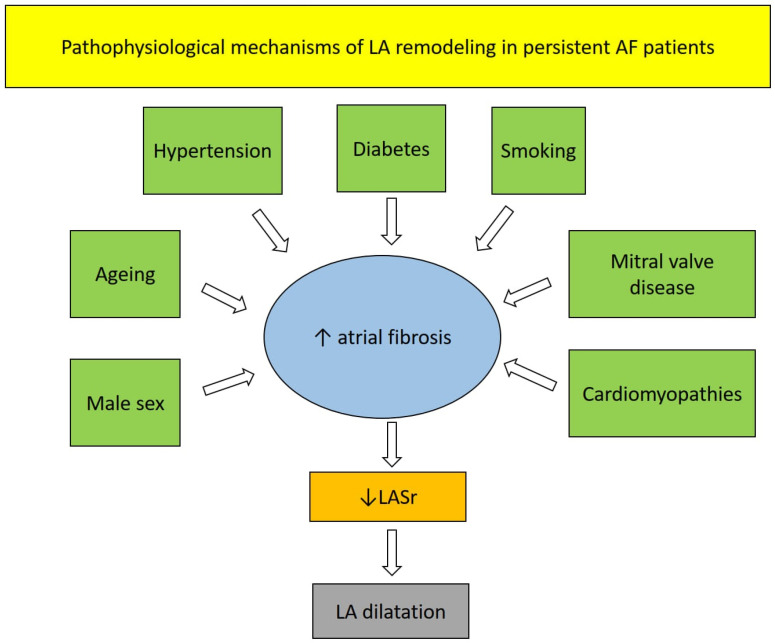
The pathophysiological mechanisms underpinning LASr impairment in persistent AF patients. AF, atrial fibrillation; LA, left atrial; LASr, left atrial reservoir strain.

LASr is a reliable surrogate of atrial fibrosis, which has been found to show a good correlation with both histology [95,96] and regional late gadolinium enhancement findings [48,97]. LASr impairment is a marker of AF-induced atrial cardiomyopathy, characterized by early interstitial collagen deposition leading to increased LA fibrosis and stiffness. These microstructural changes generally occur before LA enlargement and may be detected early by strain echocardiographic imaging [98]. Several concomitant pathophysiological conditions, such as mitral valve disease, hypertension, CAD, and cardiomyopathies may contribute to the chronicity of LV afterload elevation, causing an increase in LVFPs and leading to progressive LA dysfunction and enlargement [99,100,101] (Figure 3).

The included studies identified the amount of LA dyssynchrony, a LASr surrogate, quantified as TP-SD or dTP-LS of atrial strain, as an independent predictor of AF recurrence at 1-year follow-up [23,24,26], thus indicating that alterations in local atrial conduction properties, caused by interstitial fibrosis, are the fundamental mechanism in AF recurrence [102].

Atrial remodeling has been shown to involve simultaneously both the RA and the LA. Interestingly, Tomaselli M et al. [33] demonstrated that the patients who experienced AF recurrence had a more unfavorable RA remodeling, characterized by larger right atrial (RA) volume and lower RA strain values, compared with the patients who maintained SR. Moreover, RASr impairment was found to independently predict AF recurrence after ECV, irrespective of LASr magnitude.

AF recurrence after ECV has been previously associated also with advanced age [85], increased BMI [103], larger LAVi [104], higher E/e’ ratio [105], increased serum levels of NT-proBNP [54], previous ECV [106], and long-standing persistent AF [106].

Table 4 summarizes all the principal independent predictors of ECV failure, AF relapse after ECV, and ECV success, assessed by the included studies and scientific literature.

### 4.6. Implications for Clinical Practice

Consistent with previous studies [107,108,109], the present systematic review confirmed that 2D-STE is a feasible method for the evaluation of global and regional LA mechanics in AF patients. Due to the good agreement between GE- and Phillips-derived strain values [110,111], both vendors may provide a comparable assessment of LA mechanics. LASr measurement performed in AF was able to yield valuable prognostic information about AF relapses and the refinement of thromboembolic risk. Given the robust mechanical concordance between LA and LAA function in AF patients undergoing ECV, a possible clinical implication of this finding is to potentially avoid TEE in patients with severely impaired LA strain, reflecting advanced atrial cardiomyopathy and decreased LAA flow velocities on TEE, associated with a thromboembolic state [45,47]. These patients should be treated with rate-control therapy and oral anticoagulants. On the other hand, the AF patients who are diagnosed with normal LASr on STE examination have a significantly reduced probability of being diagnosed with LAA dysfunction on TEE. In those patients, the possibility to undergo ECV without the preliminary three to four weeks of anticoagulation could be considered in individual cases, particularly if there is no evidence of elevated thromboembolic risk score, increased natriuretic peptides, low ejection fraction, and increased LVFPs. This diagnostic approach based on LASr assessment by STE without subsequent TEE was tested by our study group during the COVID-19 pandemic as a noninvasive imaging modality for reducing the COVID-19 infection diffusion [112]. A more comprehensive approach including both LASr and RASr assessment in AF patients before ECV and a subsequent re-evaluation of their magnitude post-ECV could allow the clinicians to identify those patients who may need a closer clinical follow-up.

### 4.7. A New Method for Identifying Asymptomatic vs. Symptomatic Persistent AF Patients

**Figure 4:**
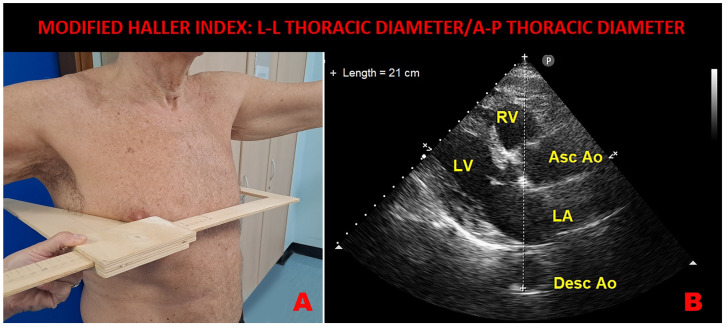
Example of modified Haller index assessment in an elderly male with circular transverse thoracic shape section, affected by asymptomatic persistent atrial fibrillation. (Panel (**A**)): L-L thoracic diameter, measured with the patient in the standing position and with open arms by using a rigid ruler in centimeters coupled to a level (the measuring device) placed at the distal third of the sternum. (Panel (**B**)): A-P thoracic diameter, obtained from the echocardiographic parasternal long-axis view, as the distance between the true apex of the sector and the posterior wall of the descending thoracic aorta, visualized behind the left atrium. Ao, aorta; A-P, antero-posterior; Asc, ascending; Desc, descending; LA; left atrium; L-L, latero-lateral; LV, left ventricle; RV, right ventricle.

Recent evidence suggests that most asymptomatic persistent AF patients scheduled for ECV are elderly males with a circular transverse thoracic shape section, higher burden of thromboembolic risk, and an echocardiographic profile characterized by hypertensive hypertrophic cardiomyopathy, moderate LA dilatation, and severe LASr attenuation [113]. Conversely, symptomatic persistent AF patients are generally younger, predominantly females, with a more concave thoracic conformation due to a narrow antero-posterior (A-P) thoracic diameter, with lower cardiovascular disease burden, and with no evidence of structural cardiomyopathy and preserved LA mechanics on echocardiographic examination [113]. Importantly, a different chest shape conformation may be predictive of the asymptomatic or the symptomatic persistent AF status. Chest shape may be noninvasively assessed by a modified Haller index (MHI), an innovative nonradiological index obtained by dividing the latero-lateral external thoracic diameter (measured by a rigid ruler coupled to a level) by the A-P thoracic diameter (measured during conventional TTE as the distance between the true apex of the sector and the posterior wall of the descending aorta, visualized behind the left atrium from the parasternal long-axis view) [114]. An MHI ≤ 2.5 and/or an A-P thoracic diameter > 18 cm, related to a more circular transverse thoracic shape section, are responsible for reduced symptom perception in asymptomatic AF patients, whereas an MHI > 2.5 and/or an A-P thoracic diameter < 13 cm, related to a concave-shaped chest wall conformation, are strongly associated with symptomatic AF status [113]. A representative example of an elderly asymptomatic persistent AF patient with a circular transverse thoracic shape section is depicted in Figure 4.

ECG Holter monitoring or implantable device (loop recorder) might preferably be considered in elderly asymptomatic males with MHI ≤ 2.5 or an A-P thoracic diameter > 18 cm, with a higher clinical score for thromboembolic risk and with increased comorbidity burden. AF patients with a severe attenuation of LA mechanics should undergo intensive cardioprotective treatment in order to prevent the future occurrence of heart failure [115,116]. Moreover, hypertension, smoking habit, sedentary life, obesity, and comorbidities should be carefully evaluated, since they play a role in disease progression and are significantly associated with worse survival [117].

### 4.8. Limitations of the Included Studies

The main limitations of the included studies were their monocentric nature, the limited number of AF patients analyzed, and the retrospective nature for 25% of them. In addition, only LAVi, LASr, and LVEF were consistently measured by the included studies, whereas the great majority of conventional and innovative echocardiographic parameters were assessed by a limited number of studies, ranging from 16.7% to 41.7% of the total. The effect of cardiovascular disease burden on LASr in AF patients was not adequately investigated by the included studies; indeed, approximately half of the studies did not mention the prevalence of smoking, dyslipidemia, TIA, and/or heart failure among AF patients. Moreover, the mean follow-up period after ECV was short (<6 months), with the consequent lack of data concerning a longer follow-up period. Even if only three studies (25% of the total) analyzed the effect of AF on LA mechanics by performing adjusted analyses for potentially confounding variables such as age and male sex, both the unadjusted and adjusted data resulted were unbiased. Finally, it is noteworthy that strain echocardiographic imaging suffers from a number of technical limitations, such as the dependence on the operator’s experience, on good image quality, on frame rate, on loading conditions, and finally, on extrinsic mechanical factors, such as the chest wall conformation [118,119,120].

## 5. Conclusions

LASr assessment before ECV may provide useful prognostic information about AF relapses and improve the refinement of the thromboembolic risk of AF patients scheduled for ECV.

Impaired LASr before ECV is a strong predictor of pro-thrombotic state, cardioversion failure, and AF recurrences over short- to mid-term follow-up.

The innovative MHI method may help clinicians identify persistent AF patients with an increased probability of severe LAS impairment, who need closer follow-up and the immediate introduction and/or uptitration of intensive cardioprotective treatment.

## Figures and Tables

**Table 1:** Summary and main findings of the included studies; 2D, two-dimensional; 3D, three-dimensional; AF, atrial fibrillation; ECV, electrical cardioversion; NS, not specified; STE, speckle tracking echocardiography.

Study Name, Publication Year, and Country	Population Size (% Males)	Mean Age (Years)	Study Design	Imaging Method	ECV Success Rate (%)	Follow-Up (Months)	AF Recurrence Rate (%)
Kaya E.B. et al. (2008), Turkey [22]	22 (36.4)	63.9	Prospective	2D-echo and 2D-STE	100	0.33	NS
Rondano E. et al. (2010), Italy [23]	130 (63.6)	67.5	Prospective	2D-echo and 2D-STE	100	12	53
Dell’Era G. et al. (2010), Italy [24]	73 (55)	71.6	Prospective	2D-echo and 2D-STE	100	1	56.1
Shaikh A.Y. et al. (2012), USA [25]	41 (70.7)	64.5	Prospective	2D-echo and 2D-STE	100	6	34
Doruchowska A. et al. (2014), Poland [26]	80 (66.2)	64.5	Prospective	2D-echo and 2D-STE	76.2	6	68.8
Costa C. et al. (2016), Spain [27]	56 (64)	67	Retrospective	2D-echo and 2D-STE	88	3	5
Cameli M. et al. (2017), Italy [28]	79 (73.4)	70.8	Prospective	2D-echo and 2D-STE	100	0	NS
Soulat-Dufour L. et al. (2019), France [29]	48 (76)	65	Prospective	3D-echo and 3D-STE	65.8	6	47.9
Sonaglioni et al. (2021), Italy [30]	125 (60)	71.5	Retrospective	2D-echo and 2D-STE	100	3	25
Arvanitis P. et al. (2022), Sweden [31]	43 (83.7)	55	Prospective	2D-echo and 2D-STE	95.3	1	9.3
von Roeder M. et al. (2022), Germany [32]	51 (71)	70	Prospective	3D-echo and 3D-STE	100	12	31
Tomaselli M. et al. (2023), Italy [33]	132 (55)	72	Retrospective	2D-echo and 2D-STE	72.6	9	22

**Table 2:** All the baseline demographic, anthropometric, clinical, laboratory, and echocardiographic parameters collected by the included studies in AF patients scheduled for ECV. The data are expressed as median and IQR. ACEi, angiotensin-converting enzyme inhibitors; AF, atrial fibrillation; A-P, antero-posterior; ARBs, angiotensin receptor blockers; BB, beta blockers; BMI, body mass index; BNP, brain natriuretic peptide; CAD, coronary artery disease; CCB, calcium channel blockers; CHA_2_DS_2_-VASc, Congestive heart failure or left ventricular dysfunction, Hypertension, Age ≥ 75 years, Diabetes, Stroke/TIA, Vascular disease, Age 65–74 years, and Sex category; CRP, C-reactive protein; EV, emptying velocity; FV, filling velocity; HF, heart failure; IQR, interquartile range; LA, left atrial; LAA, left atrial appendage; LAAT, left atral appendage thrombosis; LASr, left atrial reservoir strain; LAVi, left atrial volume indexed; LVEDD, left ventricular end-diastolic diameter; LVEDVi, left ventricular end-diastolic volume indexed; LVEF, left ventricular ejection fraction; NOACs, novel oral anticoagulants; NT-proBNP, N-terminal pro-brain natriuretic peptide; RASr, right atrial reservoir strain; RAVi, right atrial volume indexed; RWT, relative wall thickness; sPAP, systolic pulmonary artery pressure; SR, strain rate; TAPSE, tricuspid annular plane systolic excursion; TIA, transient ischemic attack; TP-SD, standard deviation of time-to-peak strain; VKAs, Vitamin K antagonists.

	Average Value (IQR)	Number of Studies for Parameters Assessed (%)
**Demographics**		
Age (years)	66.9 (55–72)	12 (100)
Male sex (%)	64.6 (36.4–83.7)	12 (100)
**Anthropometrics**		
BMI (Kg/m^2^)	28.5 (22.9–32.9)	10 (83.3)
**Cardiovascular risk factors and cardiovascular disease burden**		
Hypertension (%)	65.1 (20.9–88.7)	11 (91.7)
Smokers (%)	25.9 (20–33.6)	5 (41.7)
Dyslipidemia (%)	48.7 (22–66)	6 (50)
Type 2 diabetes (%)	19.5 (4.7–28)	10 (83.3)
CAD history (%)	19.5 (2.3–51.2)	8 (66.7)
TIA/stroke history (%)	9.9 (2.4–17.6)	6 (50)
HF history (%)	24.2 (0–41)	3 (25)
**Clinical and laboratory data**		
AF duration (months)	2.9 (0.03–5.7)	7 (58.3)
Heart rate (bpm)	97 (81–113)	5 (41.7)
CHA_2_DS_2_-Vasc score	2.6 (0.7–3.8)	7 (58.3)
BNP (pg/mL)	116 (105–205)	3 (25)
NT-proBNP (pg/mL)	1271 (1200–1409)	3 (25)
CRP (mg/dL)	0.6 (0.3–1)	3 (25)
**2D-TTE parameters**		
LA A-P diameter (mm)	45.3 (44.1–46)	5 (41.7)
LAVi (ml/m^2^)	42.9 (31.3–58.5)	10 (83.3)
LVEDD (mm)	51.2 (49–52.8)	5 (41.7)
RWT	0.40 (0.37–0.41)	3 (25)
LVEDVi (ml/m^2^)	49.5 (42.2–57)	5 (41.7)
LVEF (%)	53.5 (49.9–59.6)	12 (100)
E/e’ ratio	10.9 (9–12.9)	5 (41.7)
TAPSE (mm)	18.6 (18.2–19)	2 (16.7)
RAVi (ml/m^2^)	32.2 (29.3–35)	2 (16.7)
sPAP (mmHg)	35.4 (33–37.7)	2 (16.7)
**2D-TEE parameters**		
LAA-EV (cm/s)	40.7 (26–48.7)	3 (25)
LAA-FV (cm/s)	44.3 (32–54.7)	3 (25)
LAAT (%)	11.6 (7.1–16)	2 (16.7)
**STE indices**		
Peak systolic LASr (%)	11.4 (6.2–17.7)	12 (100)
Peak systolic LA-SR (s^−1^)	2.4 (1.65–3.2)	2 (16.7)
LA TP-SD (%)	17.5 (16.3–18.7)	2 (16.7)
Peak systolic LAA strain (%)	9.4 (9.2–9.65)	2 (16.7)
Peak systolic LAA SR (s^−1^)	2 (1.79–2.2)	2 (16.7)
Peak systolic RASr (%)	12.1 (7.2–17)	2 (16.7)
**Current medical treatment**		
VKAs/NOACs (%)	71.9 (25–92)	6 (50)
Antiplatelets (%)	27.7 (0–73.2)	4 (33.3)
ACEi/ARBs (%)	50.1 (18.6–60.6)	6 (50)
CCB (%)	32.5 (23.4–41.5)	2 (16.7)
BB (%)	47.5 (20.9–65.8)	7 (58.3)
Digoxin (%)	24.1 (9.7–51.5)	3 (25)
Amiodarone (%)	30.2 (19.5–37)	4 (33.3)
Class IC antiarrhythmic drugs (%)	32.7 (18.2–47.2)	2 (16.7)
Diuretics (%)	45.7 (33–52.8)	3 (25)
Statins (%)	34.3 (15–53.6)	2 (16.7)

**Table 3:** Quality assessment of the included studies. Q1: Was the research question or objective in this paper clearly stated? Q2: Was the study population clearly specified and defined? Q3: Was the participation rate of eligible persons at least 50%? Q4: Were all the subjects selected or recruited from the same or similar populations (including the same time period)? Were inclusion and exclusion criteria for being in the study prespecified and applied uniformly to all participants? Q5: Was a sample size justification, power description, or variance and effect estimates provided? Q6: For the analyses in this paper, were the exposure(s) of interest measured prior to the outcome(s) being measured? Q7: Was the timeframe sufficient so that one could reasonably expect to see an association between exposure and outcome if it existed? Q8: For exposures that can vary in amount or level, did the study examine different levels of the exposure as related to the outcome (e.g., categories of exposure, or exposure measured as continuous variable)? Q9: Were the exposure measures (independent variables) clearly defined, valid, reliable, and implemented consistently across all study participants? Q10: Was the exposure(s) assessed more than once over time? Q11: Were the outcome measures (dependent variables) clearly defined, valid, reliable, and implemented consistently across all study participants? Q12: Were the outcome assessors blinded to the exposure status of participants? Q13: Was loss to follow-up after baseline 20% or less? Q14: Were key potential confounding variables measured and adjusted statistically for their impact on the relationship between exposure(s) and outcome(s)? Good: met 11–14 criteria; Fair: met 6–10 criteria; Poor: met 0–5 criteria. NIH = National Institutes of Health; NS, not specified.

NIH Quality Assessment Tool for Observational Cohort and Cross-Sectional Studies															
Study Name	Q1	Q2	Q3	Q4	Q5	Q6	Q7	Q8	Q9	Q10	Q11	Q12	Q13	Q14	Quality
Kaya E.B. et al. [22]	Yes	Yes	Yes	Yes	No	Yes	Yes	No	Yes	Yes	Yes	NS	Yes	No	10 (Fair)
Rondano E. et al. [23]	Yes	Yes	Yes	Yes	No	Yes	Yes	Yes	Yes	Yes	Yes	NS	Yes	No	11 (Good)
Dell’Era G. et al. [24]	Yes	Yes	Yes	Yes	No	Yes	Yes	Yes	Yes	Yes	Yes	NS	Yes	No	11 (Good)
Shaikh A.Y. et al. [25]	Yes	Yes	Yes	Yes	No	Yes	Yes	No	Yes	Yes	Yes	NS	Yes	No	10 (Fair)
Doruchowska A. et al. [26]	Yes	Yes	Yes	Yes	No	Yes	Yes	Yes	Yes	Yes	Yes	Yes	Yes	No	12 (Good)
Costa C. et al. [27]	Yes	Yes	Yes	Yes	No	Yes	Yes	Yes	Yes	No	Yes	Yes	NS	No	10 (Fair)
Cameli M. et al. [28]	Yes	Yes	Yes	Yes	No	Yes	Yes	No	Yes	No	Yes	Yes	NS	No	9 (Fair)
Soulat-Dufour L. et al. [29]	Yes	Yes	Yes	Yes	No	Yes	Yes	No	Yes	Yes	Yes	Yes	Yes	No	11 (Good)
Sonaglioni et al. [30]	Yes	Yes	Yes	Yes	No	Yes	Yes	No	Yes	No	Yes	Yes	No	No	9 (Fair)
Arvanitis P. et al. [31]	Yes	Yes	Yes	Yes	No	Yes	Yes	No	Yes	Yes	Yes	Yes	Yes	Yes	12 (Good)
von Roeder M. et al. [32]	Yes	Yes	Yes	Yes	No	Yes	Yes	Yes	Yes	Yes	Yes	NS	Yes	Yes	12 (Good)
Tomaselli M. et al. [33]	Yes	Yes	Yes	Yes	No	Yes	Yes	No	Yes	Yes	Yes	NS	No	Yes	10 (Fair)

**Table 4:** Main independent predictors of electrical cardioversion failure, atrial fibrillation relapse after effective electrical cardioversion, and electrical cardioversion success, assessed by the included studies and scientific literature. AF, atrial fibrillation; BMI, body mass index; BSA, body surface area; dTP-LS, dispersion of time-to-peak longitudinal strain; ECV, electrical cardioversion; eGFR, estimated glomerular filtration rate; LA, left atrial; LASr, left atrial reservoir strain; LAVi, left atrial volume indexed; LV, left ventricular; NT-proBNP, N-terminal pro-brain natriuretic peptide; RASr, right atrial reservoir strain; TP-SD, standard deviation of time-to-peak strain.

**Predictors of ** **ECV failure**	Male gender, advanced age, high body weight/BSA, diabetes, low eGFR, increased NT-proBNP, CHA_2_DS_2_-VASc score > 2, LV systolic dysfunction, larger LAVi, increased E/e’ ratio, LASr impairment, previous AF history, and AF duration > 3 months.
**Predictors of** **AF relapse**	Advanced age, increased BMI, increased NT-proBNP, increased E/e’ ratio, larger LAVi, LASr impairment, large TP-SD/dTP-LS, RASr impairment, previous ECV, and long-standing persistent AF.
**Predictors of ** **ECV success**	Young age, paroxysmal AF, flutter rhythm, AF duration < 3 months, pre-treatment with antiarrhythmic agents, prior use of statins, use of biphasic waveform, normal LA size, and preserved LASr magnitude.

## Data Availability

Data extracted from the included studies will be publicly available on Zenodo (https://zenodo.org) accessed on 1 December 2023, pending acceptance by the journal.
